# Ingestion of bioactive collagen hydrolysates enhanced pressure ulcer healing in a randomized double-blind placebo-controlled clinical study

**DOI:** 10.1038/s41598-018-29831-7

**Published:** 2018-07-30

**Authors:** Fumihito Sugihara, Naoki Inoue, Sriraam Venkateswarathirukumara

**Affiliations:** 1 Nitta Gelatin, Inc., 2-22 Futamata, Yao-city, Osaka, Japan; 2 Aurous Health Care Research and Development Private Limited, No.180/109, Rangarajapuram Main Road, Kodambakkam, Chennai, 600 024 India

## Abstract

We conducted a double blind, multi-centric, placebo-controlled, randomized trial to compare the Pressure Ulcer Scale for Healing (PUSH) and Pressure Sore Status Tool (PSST) scores and wound area measurements at 16 weeks of subjects with pressure ulcers who were given standard care plus one of two types of collagen hydrolysate (CH-a), which contained low levels of prolylhydroxyproline (Pro-Hyp) and hydroxyprolylglycine (Hyp-Gly), and CH-b, which contained high levels of Pro-Hyp and Hyp-Gly) with the placebo group. A total of 120 subjects with stage II or III pressure ulcers were entered into the trial and 112 subjects completed the study. The subjects were randomized to receive CH-a (n = 39), CH-b (n = 39), or a placebo (n = 42) twice daily (10 g per day) for 16 weeks. The PUSH score, PSST score, and wound area of the CH-b group were significantly lower than the placebo group at week 16 (PUSH score, P < 0.001; PSST score, P < 0.01; wound area, P < 0.05). The PUSH score of the CH-a group was significantly lower than the placebo group at week 16 (P < 0.05). This study demonstrated that CH-b ingestion helps healing of pressure ulcers as an add-on to the standard therapy.

## Introduction

A pressure ulcer is defined as an area of localized damage to the skin and/or underlying tissue (usually over a bony prominence) caused by pressure, or pressure in combination with shear stress. Pressure ulcers develop when continuous pressure affects cellular metabolism and impedes or obstructs capillary blood flow to the skin and underlying tissue, resulting in tissue ischemia^1^. Malnutrition is an independent risk factor for the development of pressure ulcers. Appropriate nutrition is imperative for preventing and treating such wounds. Reddy M *et al*.^2^ reviewed that protein supplementation of long-term care residents improved wound healing compared with a placebo (it brought about an improvement in the Pressure Ulcer Scale for Healing [PUSH] score). For undernourished patients, supplementation with high amounts of energy and protein is recommended. In addition, various vitamins, zinc, and arginine and collagen hydrolysates should be supplied^3^.

Collagen hydrolysate (CH), which is also referred to as collagen peptides, is widely utilized as a nutritional supplement. It is a mixture of peptides of different molecular weights derived from gelatin, a form of heat-denatured collagen, via enzymatic hydrolysis. Orally ingested CH is absorbed as both free amino acids and oligopeptides, such as prolylhydroxyproline (Pro-Hyp) and hydroxyprolylglycine (Hyp-Gly), and these oligopeptides are considered to be the major factors responsible for the physiological activity of CH^4–6^.

Concerning the effects of CH on the skin, we reported that orally ingested CH containing Pro-Hyp and Hyp-Gly improved the water content, elasticity, and roughness of the skin in healthy women^7,8^. As for the effect of CH on pressure ulcers, Lee SK *et al*.^9^ reported that the combined oral administration of CH, an amino acid mixture, and the standard treatment resulted in an improvement in the PUSH score after 8 weeks’ treatment. The objective of the present study was to assess the clinical effectiveness of CH supplementation in terms of its ability to induce remission in subjects with stage II or III pressure ulcers and the safety of this approach.

## Materials and Methods

### Materials

The placebo, maltodextrin TK-16, was purchased from Matsutani Chemical Industry Co., Ltd. (Itami, Japan). Two pork skin gelatin-derived CH, which were composed of different proportions of free-form Pro-Hyp and Hyp-Gly, were used in this study. The first CH (CH-a) had a low dipeptide content (<0.01 g dipeptides per kg of product). The other CH (CH-b) had a high dipeptide content (>1 g dipeptides per kg of product). The mean molecular weights of CH-a and CH-b were 5,000 and 1,200, respectively. These products were provided by Nitta Gelatin, Inc. (Osaka, Japan), and are commercially available under the Wellnex brand. The characteristics of the test samples are shown in Table 1. Each 5-g test sample was packed in an aluminum sachet for blinding by a researcher of Nitta Gelatin Inc.

**Table 1:** Characteristics of the collagen hydrolysates (CH-a and CH-b) and placebo.

Characteristic	Unit	Placebo	CH-a	CH-b
Energy	kcal kg^−1^	3840	3860	3740
Protein	g kg^−1^	0	965	894
Carbohydrates	g kg^−1^	960	0	41
Moisture	g kg^−1^	40	33	61
Fat	g kg^−1^	0	0	0
Ash	g kg^−1^	0	2	4

### Inclusion criteria

The inclusion criteria were as follows: inpatients or outpatients of either sex who were aged between 18 and 70 years; had been diagnosed with stage II or III pressure ulcers, as defined by the National Pressure Advisory Panel of the USA (2007)^10^; had a body mass index of 18.5 to 34.9 kg m^−2^; exhibited a pressure ulcer surface area of <80 cm^2^ (multiplication of the major and minor diameters of the wound surface); were suffering from a stage II or III pressure ulcer (regardless of its location) with a PUSH (version 3.0) score of ≥5 that was likely to heal during the 6-month study period; and demonstrated moderate exudate production and a Braden score of ≥6.

### Exclusion criteria

The exclusion criteria were as follows: women who were pregnant or lactating; women of childbearing potential who were not taking adequate contraceptive measures; patients with stage IV pressure ulcers; patients that were being fed via a tube; patients with diabetic foot ulcers; patients who received immunotherapy or cytotoxic chemotherapy within the 60 days before enrollment; patients who had taken systemic steroids within the 30 days prior to enrollment; patients that had received topical therapy other than steroidal therapy during the 7 days prior to enrollment; patients who were human immunodeficiency virus-, hepatitis B virus-, or hepatitis C virus-positive; patients with pre-existing demyelinating disorders; patients with hepatic, renal, or metabolic disease that was likely to interfere with their participation in or completion of the study; patients with arterial or venous disorders that had the potential to cause ulcerated wounds; patients with a history of established diabetes mellitus and a fasting blood glucose level of >200 mg dL^−1^; patients with any condition that would interfere with wound healing (e.g., a connective tissue disorder, immunological disorder, or clinical obesity); patients who were malnourished; patients with wounds caused by malignancy; patients with burns or scalds; patients who had used any form of complimentary alternative medicine in the preceding 2 months; patients that were known to exhibit hypersensitivity reactions to protein products; patients with any dermatological condition or disorder that might interfere with the appropriate assessment or treatment of ulcers; current smokers; patients who had participated in any other clinical study during the 3 months prior to this trial; patients who were unwilling or unable to comply with the study procedures; patients who were considered to be unsuitable candidates by the investigator for any reason.

### Study design

This was a 16-week double-blind, multi-centric, placebo-controlled, randomized clinical study that aimed to determine the effectiveness, safety, and tolerability of CH (CH-a or CH-b) as an add-on nutritional supplement during the management of subjects with stage II or III pressure ulcers.

The subjects were enrolled according to the inclusion and exclusion criteria. After enrollment, a centralized allocation method was used to assign the study subjects to the CH-a, CH-b, or placebo group. The subjects were allocated independently to each group using computer-generated randomization code and the SAS® software by a doctor of Aurous Health Care Research and Development Private Limited. The trial sites were also subjected to centralized allocation. The randomization was balanced, and access to the code was strictly controlled. The study protocol, informed consent documents, and case reports, along with all of the secondary documents used for the study were reviewed and approved by an independent ethics committee (India National Ethics Committee, Approved date: December 19^th^, 2009). The study was conducted in accordance with the ethical principles laid out in the current version of the Declaration of Helsinki and the ICH-GCP guidelines and was registered (No. CTRI/2009/091/001097, Registration date: April 15^th^, 2010) with the Clinical Trial Registry, India. Written informed consent was obtained from all subjects who participated in this study. The subjects were advised to orally consume 5 g of CH or the placebo, dissolved in 250 ml water or milk in the morning and night after eating food. Regarding the standard therapy, the subjects were treated with antimicrobials, antiseptics, wound debridement, and wound dressing, as required.

**Figure 1:**
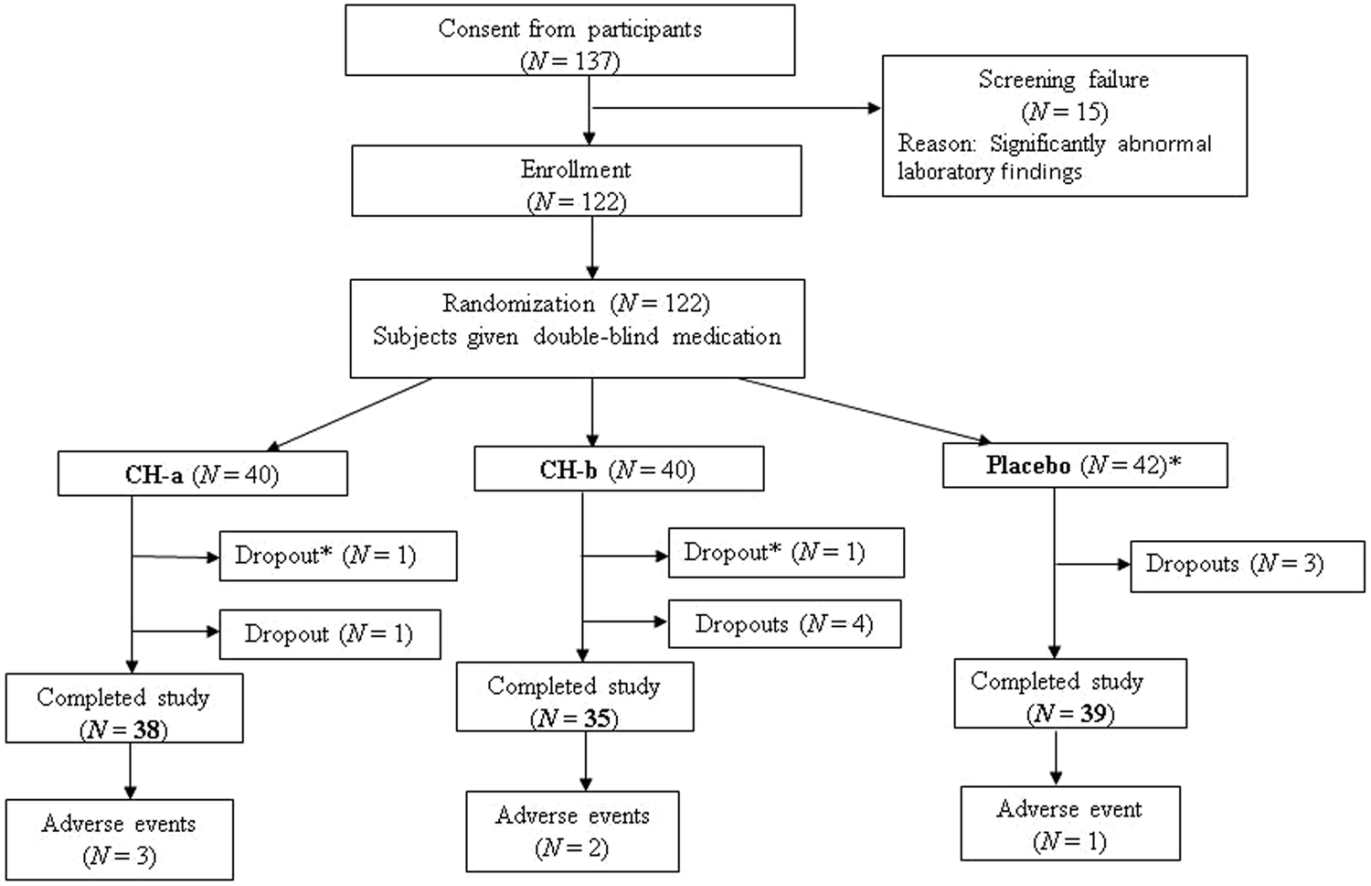
Flow chart of the study design and the passage of the subjects through it. After enrollment, 122 subjects were randomized into three arms, the CH-a, CH-b, and placebo groups. Thirty-eight subjects in the CH-a group, 35 subjects in the CH-b group, and 39 subjects in the placebo group completed the study. *One subject each from the CH-a and CH-b groups, who did not satisfy the test criteria as they dropped out before any post-baseline data had been obtained, were replaced with two further subjects, who were then subjected to randomization.

A total of 137 subjects were screened using the inclusion and exclusion criteria. One hundred and twenty subjects were considered to be eligible for the study based on these criteria and so were enrolled. After enrollment, the subjects were randomized in a 1:1:1 ratio to receive CH-a, CH-b, or the placebo, as illustrated in Fig. 1. After the first visit (week 0), two subjects (one each from the CH-a and CH-b groups) dropped out citing travel difficulties. Hence, two more subjects were enrolled in the study. They were allotted to the placebo arm using the same randomization procedure. Eight more subjects subsequently dropped out of the study, but they were not replaced as at least one set of post-baseline data had been obtained for them. Therefore, a total of 112 subjects completed the study and were analyzed.

The variables used to assess the efficacy of the treatments included the PUSH score, the Pressure Score Status Tool (PSST) score as first outcomes, and wound area according to photographic measurements as a second outcome (length and breadth were measured in cm using a metric ruler, and the resultant area values, length x width, were recorded in cm^2^).

The primary treatment efficacy endpoint was to compare CH group and placebo group for PUSH score, PSST score and wound area at week 16. The secondary treatment efficacy endpoint was the following 2 steps: (1) the primary improvement was a reduction in the PUSH score of ≥5 points and a reduction in the PSST score of ≥10 points between the baseline and week 16, and (2) the secondary improvement was a reduction in the PUSH score of 3–4 points and a reduction in the PSST score of 5–9 points between the baseline and week 16.

Clinical laboratory and biochemical evaluations, including blood and urine analyses, were used to assess the safety of CH-a and CH-b. Vital signs, such as temperature, pulse rate, respiratory rate, and systolic and diastolic blood pressure, and other parameters related to safety were analyzed.

### PUSH Score Calculation

The PUSH Score was calculated as per the PUSH Table.

1. The following three individual variables were measured to calculate the PUSH Score: (1) Length X Width: The greatest length and the greatest width of the wound were measured in square centimetres. The two values were multiplied and corresponding score from the PUSH table (An instrument to measure healing, Version 3.0:98’ National Pressure Ulcer Advisory Panel) was noted as the first variable; (2) Exudate Amount: The amount of exudate, post removal of dressing and before the application of any topical agent was estimated. The corresponding score from the PUSH table was noted as the second variable.; (3) Tissue Type: The tissue of the wound was examined. The corresponding score from the PUSH table was noted as the third variable.
2. These three variables were added to get the PUSH Score.
3. The PUSH score was calculated as baseline and periodically upon treatment to show improvement metrics.

### PSST Score Calculation

The PSST Score was calculated based on the assessment of 13 different variables as follows: Size, Depth, Edges, Undermining, Necrotic Tissue Type, Necrotic Tissue Amount, Exudate Type, Exudate, Skin Color Surrounding Wound, Peripheral Tissue Edema, Peripheral Tissue Indurations, Granulation Tissue, Epithelialisation.

1. Each of the 13 variables has 5 choices that the assessor could choose from, based on the nature and presentation of wound on the day of examination.
2. The choices ranked from Score 1 to Score 5.
3. The assessor marked a score corresponding to the presentation for each of the 13 variables.
4. The sum total of all the scores gave the PSST Score.

### Statistical analyses

The Kolmogorov-Smirnov Z test was performed to assess the normality of the PUSH score, PSST score, and wound area data. For normally distributed data, two-way ANOVA was used during comparisons between groups (the CH-a, CH-b, and placebo groups), and repeated-measures ANOVA was employed for intra-group comparisons. Comparisons between the baseline and week-16 data were conducted using the paired *t*-test. ANCOVA was used to adjust for baseline differences among the treatment groups. For non-normal data, the Kruskal-Wallis and Friedman tests were used for intergroup and intra-group comparisons, and comparisons between the baseline and week-16 data were carried out with the Wilcoxon signed rank test. Evaluations of the proportions of subjects who exhibited improvements in their PUSH scores, PSST scores, or wound area were conducted using the chi square test or Fisher’s exact test.

## Results

The demographic characteristics of the subjects are shown in Table 2. All of the subjects were advised to follow their normal diet; i.e., the diet they were consuming at the time of their inclusion in the clinical study. None of the patients involved in the clinical study had any problems with their diet. Finally, each group CH-a 38, CH-b 35, placebo 39 subjects were analyzed (Fig. 1).

**Table 2:** Demographic data of the subjects selected for the clinical study.

Variable	Group		
	Placebo (*N* = 39)	CH-a (*N* = 38)	CH-b (*N* = 35)
Age (years)	46.4 (11.1)	38.0 (8.4)	45.1 (12.1)
**Sex**			
Male	17	25	20
Female	22	13	15
Height (cm)	154 (9)	157 (8)	153 (10)
Weight (kg)	62.1 (6.8)	64.5 (6.9)	60.6 (7.2)
BMI (kg m^−2^)	26.4 (3.3)	26.4 (3.4)	25.8 (2.9)
PUSH score (points)	11.92 (1.90)	12.00 (1.51)	12.34 (1.92)
PSST score (points)	29.79 (2.31)	29.71 (2.31)	30.20 (1.69)
Wound area (cm^2^)	12.85 (10.29)	12.16 (9.22)	13.23 (9.56)

Data are expressed as mean (standard deviation) values.

PUSH: pressure ulcer scale for healing, PSST: pressure score status tool.

### Efficacy

**Figure 2:**
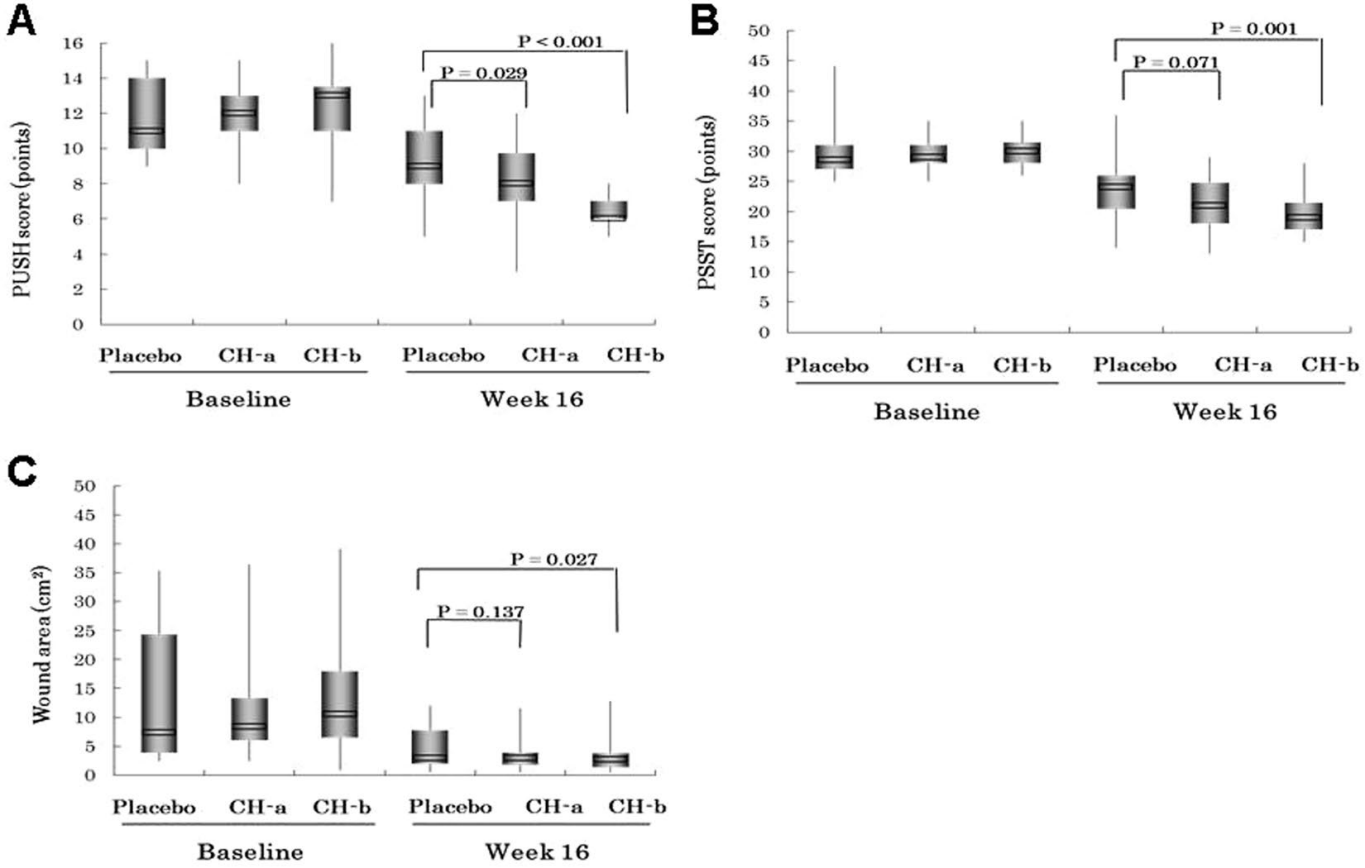
Comparisons of the primary treatment efficacy parameters (the Pressure Ulcer Scale for Healing (PUSH) score (**A**), Pressure Sore Status Tool (PSST) score (**B**), and wound area (**C**)) between the baseline and week 16. Data are expressed as mean values (*N* = 38, 35, and 39 for the CH-a, CH-b and placebo groups, respectively). The PUSH and PSST scores and the pressure ulcer surface area of the CH-b group were significantly lower than those of the placebo group at week 16 (PUSH score, *P* < 0.01; PSST score, *P* = 0.01; wound area, *P* = 0.027). The PUSH score of the CH-a group was significantly lower than that of the placebo group at week 16 (*P* = 0.029).

The changes in the patients’ PUSH scores, PSST scores, and wound area measurements are shown in Fig. 2. The PUSH score, PSST score, and wound area values of the CH-b group were significantly lower than those of the placebo group at week 16 (PUSH score: 6.46 ± 0.98 vs. 9.26 ± 2.09, P < 0.01; PSST score: 19.71 ± 3.08 vs. 23.38 ± 3.85, P = 0.01; wound area: 3.19 ± 2.88 vs. 5.00 ± 3.88, P = 0.027). The PUSH score of the CH-a group was significantly lower than that of the placebo group at week 16 (8.21 ± 2.04 vs. 9.26 ± 2.09, P = 0.029).

These findings agreed with the response rates of each group. Specifically, >71% of the subjects in the CH-b group exhibited improvements in their PUSH and PSST scores, while no such improvements were seen in the CH-a or placebo groups, as shown in Table 3.

**Table 3:** Frequencies of each treatment efficacy improvement.

Efficacy improvement*	PUSH score			PSST score		
	Placebo (*N* = 39)	CH-a (*N* = 38)	CH-b (*N* = 35)	Placebo (*N* = 39)	CH-a (*N* = 38)	CH-b (*N* = 35)
Primary (%)	9.5	18.0	28.2	7.1	10.3	23.1
Secondary (%)	11.9	28.2	43.6	11.9	38.5	51.3
Primary + secondary (%)	21.4	46.2	71.8	19.0	48.8	74.4

^*^The primary treatment efficacy improvement was a reduction in the PUSH score of ≥5 points and a reduction in the PSST score of ≥10 points between the baseline and week 16.

The secondary treatment efficacy improvement was a reduction in the PUSH score of 3–4 points and a reduction in the PSST score of 5–9 points between the baseline and week 16.

### Biochemical evaluations

As part of the safety assessment, laboratory analyses of various serological and urinary biochemical parameters were performed. The baseline and week 16 data are shown in Table 4. Although many biochemical parameters differed significantly between the baseline and week 16, there were no clinically significant abnormalities. These findings demonstrated the safety of CH-a and CH-b in humans. Moreover, the US Food and Drug Administration (US FDA) has classified gelatin and CH as Generally Recognized as Safe (GRAS) products.

**Table 4:** Results of the biochemical evaluations.

Biochemical analysis	Units	Placebo (*N* = 39)		CH-a (*N* = 38)		CH-b (*N* = 35)	
		Baseline	Week 16	Baseline	Week 16	Baseline	Week 16
Blood urea	μmol L^−1^	9.5 ± 6.3	12.7 ± 6.5^#^	7.9 ± 2.4	13.4 ± 4.0^#^	8.3 ± 3.2	12.0 ± 3.7^#^
Uric acid	μmol L^−1^	267.3 ± 79.2	342.8 ± 85.9^#^	304.4 ± 84.8	328.0 ± 88.5^#^	281.3 ± 82.0	328.0 ± 88.5^#^
Serum creatinine	μmol L^−1^	86.3 ± 113.1	75.3 ±± 14.3	71.4 ± 12.7	78.0 ± 20.0	69.1 ± 13.1	73.8 ± 16.4
Total bilirubin	μmol L^−1^	9.8 ± 5.5	12.8 ± 5.2^#^	14.1 ±± 8.7	11.3 ± 5.8	11.9 ± 5.9	15.3 ± 15.4
Direct bilirubin	μmol L^−1^	2.1 ± 0.8	2.8 ± 2.3^#^	2.4 ± 1.2	3.1 ± 3.0	2.4 ± 1.0	3.1 ± 2.7
SGOT	u L^−1^	23.4 ± 8.4	29.6 ± 7.5^#^	24.2 ± 6.9	31.3 ±± 5.5^#^	23.7 ± 7.9	29.3 ± 7.6^#^
SGPT	u L^−1^	20.7 ± 8.6	30.1 ± 8.5^#^	22.7 ± 14.8	34.3 ± 8.6^#^*	29.6 ± 37.5	32.3 ± 9.8
ALP	u L^−1^	148.6 ± 57.0	197.2 ± 46.1^#^	146.8 ± 42.2	185.0 ± 52.9^#^	157.7 ± 53.3	185.0 ± 52.9^#^
Random blood glucose	mmol L^−1^	5.9 ± 1.3	7.0 ± 1.6^#^	5.6 ± 1.2	7.1 ± 1.5^#^	6.3 ± 2.7	6.9 ± 2.3
Total cholesterol	mmol L^−1^	4.6 ± 0.8	4.4 ± 0.9	4.6 ± 0.9	4.5 ± 0.7	4.4 ± 0.8	4.3 ± 0.9
Total protein	g L^−1^	72.0 ± 4.3	73.4 ± 6.5	71.3 ± 11.0	75.3 ± 12.8^#^*	72.4 ± 5.6	74.4 ± 9.0
Serum albumin	g L^−1^	41.9 ± 6.4	43.1 ± 5.9	41.8 ± 5.7	47.8 ± 6.5^#^*	42.1 ± 6.0	45.2 ± 7.5^#^

Data are expressed as mean ± SD values.

^#^P < 0.05 vs. baseline; *P < 0.05 vs. placebo.

SGOT, serum glutamic oxaloacetate; SGPT, serum glutamic pyruvate transaminase; ALP, alkaline phosphatase.

As an indicator of the subjects’ nutritional status, we focused on the serum albumin level. Compared with those seen at the baseline, the serum albumin levels of the CH-a and CH-b groups were significantly increased at week 16, indicating that the nutritional statuses of these two groups had significantly improved. Furthermore, the serum albumin levels of the CH-a group and placebo group differed significantly at week 16, indicating that the nutritional status of the CH-a group was significantly better than that of the placebo group at week 16. On the other hand, in the placebo group there was no significant increase in the serum albumin level, and hence, the patients’ nutritional status did not significantly improve during the study period.

### Adverse events

Among the 39 subjects treated with CH-a, two experienced moderate constipation, and one suffered mild diarrhea. Of the 39 subjects who received CH-b, two developed moderate diarrhea, and one of the 42 subjects treated with the placebo experienced a mild headache. It is possible that all of these adverse events were caused by the administered treatment/placebo. However, they only persisted for one day and were resolved with concomitant medication. Upon re-challenge, none of the adverse events reappeared.

## Discussion

The present study demonstrated that the oral ingestion of CH-b, which contains higher concentrations of the free-form bioactive peptides Pro-Hyp and Hyp-Gly, resulted in significantly greater improvements in the PUSH score, PSST score, and wound area compared with the ingestion of a placebo. Moreover, the ingestion of CH-a, which contains lower concentrations of these bioactive peptides, produced significantly greater improvements in the PUSH score than the placebo. This effect of CH-a was supported by the previous finding that the ingestion of CH together with an amino acid mixture led to a significant improvement in the rate of change in the PUSH score within 8 weeks^9^. These results suggest that although all CH are derived from similar raw materials, it might be possible to control the healing effects of CH on pressure ulcers by altering their dipeptide content, e.g., by modifying the processes used to produce CH.

Although the examined CH and placebo contained similar amounts of energy (Table 1), it is considered that the improvements in the nutritional status of the patients seen in the CH-a and CH-b groups at week 16 contributed to the improved healing of their pressure ulcers.

Good nutrition definitely plays an important role in wound healing^2^. However, nutrition doesn’t induce the production of extracellular matrices which are the key in tissue repair. Moreover the indication for this clinical study is Pressure Ulcer which is usually a resistant wound to many therapies available currently. The direct supplementation of safe and oral CH, especially CH-b which contains higher concentrations of Pro-Hyp and Hyp-Gly, will definitely boost the extent of repair and significantly reduce the time taken for wound healing. This is what we like to claim as important and special in the treatment of pressure ulcers.

The significant improvements in the PUSH and PSST scores and wound area induced by CH-b can be attributed to the physiological functions of the oligopeptides absorbed into the subjects’ blood. We previously reported that large amounts of Pro-Hyp and Hyp-Gly were absorbed into the blood after the ingestion of CH-b by healthy subjects^11^. Furthermore, in an experimental study in which [^14^C]-Pro-Hyp was orally administered to rats it was reported that the Pro-Hyp reached the skin within 30 minutes^12^. Pro-Hyp and Hyp-Gly increased the proliferation of fibroblasts that had migrated from skin pieces on a collagen gel culture system^5^, and Pro-Hyp promoted hyaluronan synthase 2 mRNA expression and hyaluronic acid synthesis by hyaluronan synthase 2 in cultured skin fibroblasts^13^. Furthermore, an experimental study involving a pressure ulcer rat model reported that Pro-Hyp and Hyp-Gly were absorbed into the blood in the CH oral administration group, and the wound area ratio was significantly lower in this group than in the control group^14^. These findings suggest that the Pro-Hyp and Hyp-Gly absorbed into the blood after the ingestion of CH act on fibroblasts in the dermal layers of pressure ulcers and also might affect stem cells^15^, resulting in re-epithelialization and improved healing.

In order to confirm that the ingestion of CH promotes the healing of pressure ulcers, it will be necessary to (1) elucidate the mechanism responsible for the accelerated pressure ulcer-healing induced by oligopeptides, such as CH-derived Pro-Hyp and Hyp-Gly, and (2) carry out a large-scale double-blind study.
